# The Role of the Pre-B Cell Receptor in B Cell Development, Repertoire Selection, and Tolerance

**DOI:** 10.3389/fimmu.2018.02423

**Published:** 2018-11-15

**Authors:** Thomas H. Winkler, Inga-Lill Mårtensson

**Affiliations:** ^1^Chair of Genetics, Department of Biology, Nikolaus-Fiebiger-Center for Molecular Medicine, Friedrich-Alexander-University Erlangen-Nuremberg, Erlangen, Germany; ^2^Department of Rheumatology and Inflammation Research, Institute of Medicine, The Sahlgrenska Academy, University of Gothenburg, Gothenburg, Sweden

**Keywords:** surrogate light chain, pre-B cells, B-cell development, allelic exclusion, VpreB, λ-5

## Abstract

Around four decades ago, it had been observed that there were cell lines as well as cells in the fetal liver that expressed antibody μ heavy (μH) chains in the apparent absence of *bona fide* light chains. It was thus possible that these cells expressed another molecule(s), that assembled with μH chains. The ensuing studies led to the discovery of the pre-B cell receptor (pre-BCR), which is assembled from Ig μH and surrogate light (SL) chains, together with the signaling molecules Igα and β. It is expressed on a fraction of pro-B (pre-BI) cells and most large pre-B(II) cells, and has been implicated in IgH chain allelic exclusion and down-regulation of the recombination machinery, assessment of the expressed μH chains and shaping the IgH repertoire, transition from the pro-B to pre-B stage, pre-B cell expansion, and cessation.

## The genes encoding SL chain

In the late 70's, it was shown that certain cell lines and cells in the fetal liver expressed antibody μ heavy (μH) chains in the absence of *bona fide* light chains (1, 2) which was surprising considering that μH chains by themselves might be toxic to the cell. A few years thereafter, a gene termed λ5 was cloned in mice (3), which showed homology to the constant region of Ig λL chains, Cλ1–4, hence the fifth. However, by contrast to Ig λL (and κL) chains, λ5 did not undergo recombination. Around that time a molecule termed omega was shown to associate with μH chains in pre-B but not B cell lines (4), and it was suggested that this might function as a “surrogate” for *bona fide* IgL chains, and “may well prove to be the product of the λ5 gene.” Subsequently it was indeed found to be the case. Anyhow, examining the λ5 gene in more detail it was clear that exons 2 and 3 showed homology to J and C of bona fide λ light chains whereas exon 1 did not show homology to Ig or any other known protein (5). It was thus unclear whether a variable-like gene or gene segment was missing. Soon thereafter, the VpreB1 and VpreB2 genes were cloned (6). The two genes are 97% identical, and did indeed show homology to Ig V gene segments in exon 1 whereas exon 2 did not show homology to Ig or any other known protein. It was later on shown that both VpreB genes are transcribed, although VpreB2 is expressed at lower levels than VpreB1 (7). The human counterpart, VPREB1 was cloned soon thereafter of which there is only one in the genome (8), and it turned out that IGLL1 (λ5) had already been cloned (14.1) (9, 10). There are two additional IGLL1, 16.1, and 16.2, which are pseudogenes though seemingly used as templates in a process termed gene conversion (11). The genes encoding surrogate light (SL) chain are located on the same chromosome as Ig λL chains, on chromosome 16 and 22, in mice and humans, respectively. In mice, VpreB1 and λ5 are located 4–5 kb apart, whereas VpreB2 is located approximately 1 Mb downstream of λ5 and around 1 Mb upstream of the λL locus. The organization of these genes in humans is quite different in that VPREB1 is located within the λL V gene segments whereas IGLL1 (14.1, 16.1, and 16.2) is located downstream of C_λ_7. For simplicity, the genes in both mice and humans are hereafter termed VpreB1 and λ5.

## The pre-BCR complex

**Figure 1:**
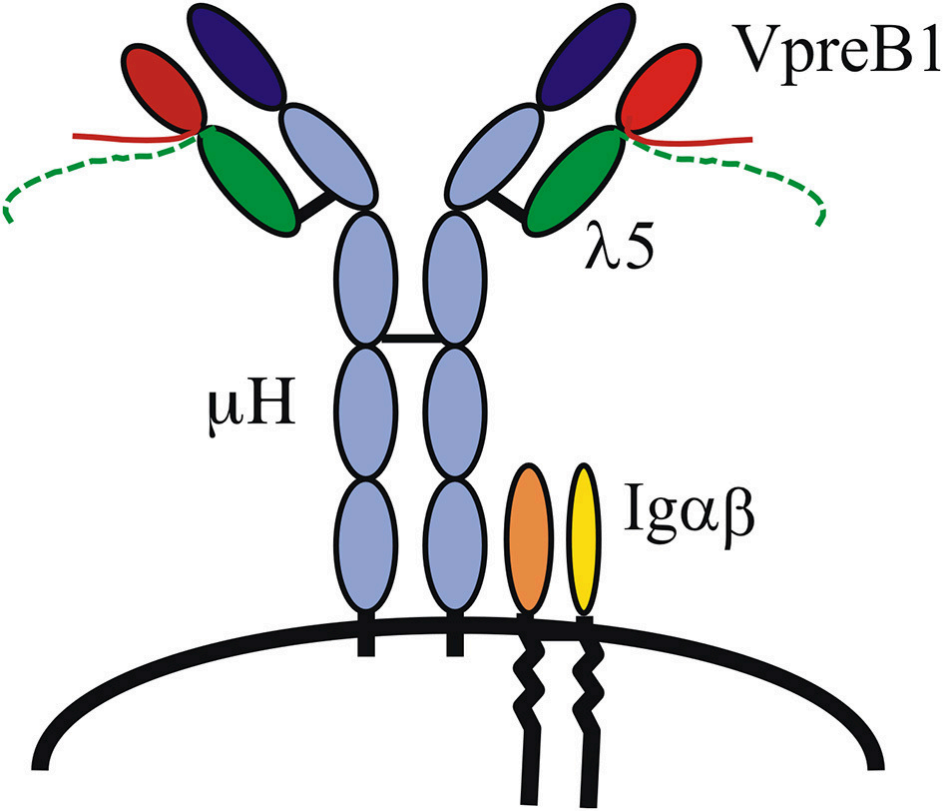
The pre-B cell receptor (pre-BCR). A pre-BCR is assembled from antibody heavy (μH) and surrogate light chains together with the signaling molecules Igα and Igβ. The SL chain is composed of VpreB1/2 and λ5. VpreB and λ5 each contains a unique region, depicted as tails protruding from the respective molecule.

That the VpreB1 and λ5 genes encode the SL chain and did indeed form a complex with μH chains was demonstrated by several groups, and it was also shown that the signaling molecules Igα and β were part of the complex and necessary for pre-B cell receptor (pre-BCR)-mediated signaling (Figure 1) (12, 13). As mentioned, the VpreB and λ5 genes show homology to IgL chains, V_λ_ and J–C_λ_, respectively, and each gene also encodes a unique region (UR). The VpreB-UR is encoded by the second exon and results in a tail of around 20 amino acid (aa) residues, and the λ5-UR is encoded by the first exon and results in a tail of ~ 50 aa. Both URs are unusual in that they contain a high proportion of charged residues, the VpreB-UR contains several negatively charged and the λ5-UR several positively charged aa residues of which most are arginine. Proper folding and stabilization of SL chain require the URs as well as the extra beta-strand in λ5 (14). Structure analyses of a mouse pre-BCR using NMR suggested that the two URs meet and protrude where the CDR3 of L chains would be located in a BCR (15) (Figure 1). This as well as the importance of the extra beta-strand in IGLL1 was confirmed after crystallization of a human pre-BCR (16), although most of the two URs were removed in order to crystallize the complex. Nevertheless, this study also suggested that a pre-BCR resembles a BCR with the exception of the URs that appear to protrude from the complex. The latter has implications in that it indicates that the pre-BCR might bind one or more ligand(s). Additional NMR studies have shown that the human λ5-UR displays a helical structure (15) and binds to galectin-1 (17).

## A leaky phenotype of SL chain deficient mice

After the discovery of the SL proteins and genes, as discussed above, the question was, what the function of such a SL chain would be during B cell development. With the advent of gene targeting in embryonic stem cells (18) and the first germline transmission of the targeted cells to generate knockout mice (19) gene targeting was the first choice to illuminate the function of the SL chain in the mouse. Already in 1992, Kitamura et al. published the analysis of the λ5 knockout (λ5T) mouse (20). The λ5T mouse was among the first 50 knockout mice ever created, illustrating the interest in the function of the SL chain at the time. The phenotype of the mice was surprising to the authors as B cell numbers and frequencies were reduced in the mutant mice but B lymphocytes were clearly present and serum immunoglobulin levels reached almost normal levels (20). Moreover, later on the genes of the other component of the SL chain were mutated, namely *Vpreb1* and *Vpreb2*, where *VpreB1/VpreB2* double-mutant mice displayed a phenotype very similar to λ5T mice (21). Targeting the two separately demonstrated a slight reduction in pre-B cells in mice lacking *VpreB1* but not in those lacking *VpreB2*, presumably due to the lower expression levels of the latter (22, 23). The complete deletion of λ5, VpreB1, and VpreB2 resulted in no additional phenotype regarding B cell numbers (24).

In the year before, Kitamura et al. used a knockout mouse model in which the membrane part of μH chain was deleted to show that expression of a membrane bound μH chain is absolutely essential for the development of B lymphocytes (25). Likewise, mice with targeted mutations in the *Rag-1* or *Rag-2* genes, unable to perform VDJ recombination and therefore unable to express a μHC protein, had no detectable mature B cells in the lymphoid organs (26, 27). This had been published just 2 months before the publication of the λ5T mouse. As the signaling capacity of the pre-BCR as well as the BCR was believed to be dependent on Igα and Igβ (28, 29), it was not surprising that *B29*/*Ig*β mutant mice also lacked B cells in the peripheral lymphoid organs (30). In addition, it was later shown that Igα and Igβ are not redundant in their function, as also Igα deficient mice lack detectable B cells (31). In light of the complete absence of B cells in these knockout mice with defects in the formation, the membrane deposition or signaling capacity of the preBCR, the incomplete phenotype of λ5T mice was puzzling and the phenotype was called “leaky” (20), perhaps referring to the leaky phenotype of *scid* mice (32). Clearly, much had to be learned about B cell development at the time. Over the next 4–5 years, several laboratories, including that of Ton Rolink and Fritz Melchers at the Basel Institute of Immunology were involved in unraveling the cellular and molecular processes of B cell development, and in particular the role of the SL chain. During this time, the phenotype of the pre-Tα (part of the pre-T cell receptor) knockout mice was published with an astonishingly similar phenotype to that of the λ5T mice in the development of α/β T cells (33). T cell development is strongly impaired but mature α/β T cells do develop.

## Understanding the function of the pre-B cell receptor in b cell development

The earliest understanding of the coordinated development of B lymphocytes according to the rearrangement status of the immunoglobulin genes was derived from the analysis of Abelson virus-transformed pro- and pre-B cell lines. In a seminal paper published 1984 by Alt and colleagues the ordered rearrangement model of B cell development was proposed (34). The positive regulatory role of the μH chain on progression in differentiation was directly shown in transformed B cell lines that sequentially undergo Ig rearrangements in cell culture (35). The experiments with transformed cell lines suggested that D to J_H_ precede V_H_ to DJ rearrangements in the IgH locus and IgH rearrangement precedes that of IgL. In addition, the experiments by Reth et al. on transformed pre-B cell lines suggested that a successfully rearranged IgH, i.e., encoding a μH chain protein, directly mediates differentiation as well as recombination of the IgL loci (35). This apparently strict dependency on μHC expression for IgL recombination was later debated in the context of explaining the leaky phenotype of the λ5T mouse. Ehlich et al. had shown that the IgH and IgL loci rearrange independently at early stages of B cell development (36). In the laboratory of Rolink and Melchers, IgκL recombination independent of μH chain expression was shown in IL-7-cultured pre-B cell lines and clones (37), but IgL rearrangement only occurred after differentiation into small pre-B cells.

Several different models for B cell development were proposed in 1991 and used by different laboratories over the coming years (38–40). The scheme proposed by Osmond (39) used the expression of the TdT and μH chain proteins in combination with B220-positivity to distinguish three μH chain negative pro-B cell stages, large cycling and small resting pre-B cells expressing intracellular μHC (icμHC) and resting B cells expressing surface μH chain (IgM) (39). Hardy et al. described several early pro-B cell subpopulations with the help of the cell surface markers B220, CD43, BP-1(CD249), and heat-stable antigen (HSA, CD24) (38), resulting in three fractions A, B, and C that were cμHC negative. CD43-negative pre-B cells were termed fraction D and surface IgM positive cells fraction E and fraction F, the latter co-expressing IgD. The clear advantage of this characterization as opposed to the intracellular markers used by Osmond was the possibility to separate living cells by FACS was possible for further analysis, e.g., in *in vitro* culture systems and for RNA/DNA analyses. The scheme proposed by Rolink and Melchers (40), finally, focused entirely on the Ig rearrangement status of the B cell progenitors and precursors for their nomenclature. B cell progenitors without rearrangements in the Ig loci were named pro-B cells, DJ_H_-rearranged cells were named pre-BI and those with a V_H_DJ_H_-rearrangement were named pre-BII cells. SL chain expression was found in pro-B, pre-BI and pre-BII cells but not in IgM surface positive B cells (40). Interestingly, none of the schemes at that time had a clear idea at which stages exactly the pre-BCR would be expressed, which was described on the cell surface of cell lines by Tsubata and Reth (13).

A break-through for further insights into the role of the SL chain in B cell development was when the Rolink and Melchers laboratory discovered two surface markers whose expression matched the μHC-negative and μHC-positive stages of pro-B and pre-B cells, respectively. The membrane tyrosine kinase c-kit (CD117) is expressed on μHC-negative pro-B cells in the BM (41) and CD25 on μH chain positive pre-B cells (42). The latter publication has 222 citations until today. With the help of monoclonal antibodies against the SL chain Karasuyama et al. showed that the SL chain is expressed on cycling cells which are μH chain negative and are also present in Rag-deficient mice, i.e., on pro-B cells (43). This correlated with RNA-expression data published before, showing that λ5 and VpreB1 are expressed in fractions B and C according to the nomenclature of Hardy (44). Both publications agreed that SL chain expression is confined to cycling cells at early stages of B cell development in the mouse (44, 43). This was in contrast to findings by the group of Max Cooper analyzing human pre-B cell development and describing surface pre-BCR expression at late stages of pre-B cell development (45). Whereas it was still not possible to detect the pre-BCR on the surface in mice, in humans a weak surface expression was inevitably shown by Lassoued et al. (45), confirming the potential signaling function proposed by Tsubata and Reth (13). With yet another monoclonal antibody shown to specifically bind to an epitope formed by the μHC in complex with the SL chain it was finally possible to detect the pre-BCR on the cell surface of *ex vivo* isolated mouse bone marrow B lymphocytes (46). Two different complexes containing the SL chain were detected on pro- and pre-B cells isolated from the bone marrow. One complex present on all c-kit positive pro-B cells consisted of λ5 and VpreB1 but not μHC. As these were pro-B cells, the complex was termed a pro-BCR, a receptor that in addition to SL chain consists of several molecules, of which only one has been characterized, BILL-Cadherin/cadherin-17 (47). Also, human pro-B cells express a pro-BCR (48).The other complex, as detected by the pre-BCR specific antibody SL156 was present on a small subpopulation of extremely large and cycling pre-B cells at the transition of pro-B and pre-B cells. These cells have downregulated c-kit almost entirely and express CD25 as a marker for pre-B cells (46). These finding not only reconciled the discrepancies between human and mouse SL chain expression but also placed the pre-BCR expression at a heavily cycling stage of B cell developmental at the transition from the pro-B to pre-B cell stage.

**Figure 2:**
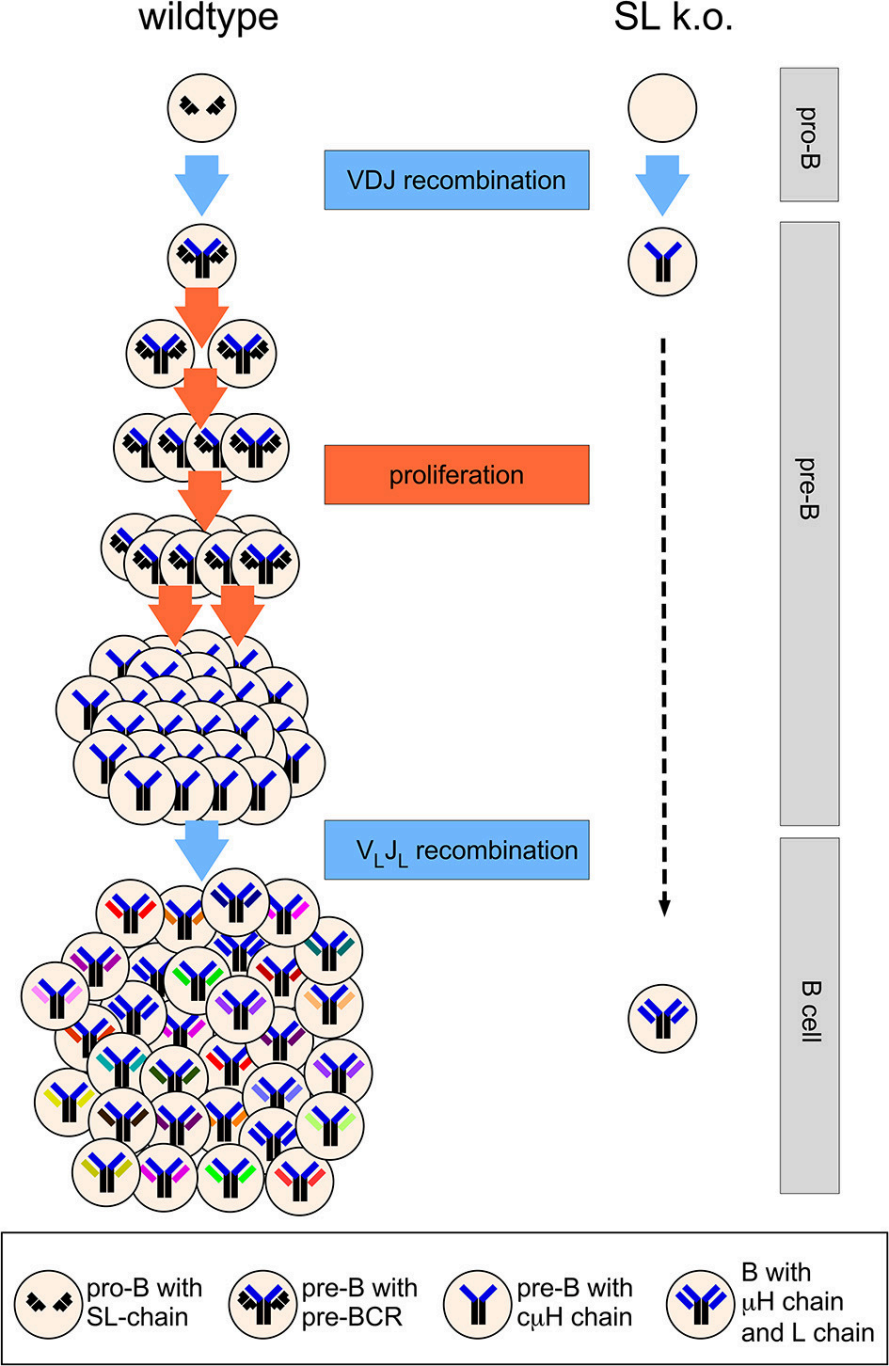
Simplified scheme of B cell development in wild type and SL chain knockout mice. At the pro-B cell stage, several cell divisions take place (not shown) and both alleles of the IgH locus will finally become DJ_H_-rearranged. A functional V_H_DJ_H_ rearrangement will code for a μH chain assembling with the SL chain. The pre-BCR induces proliferative expansion of pre-B cells, which is missing in SL chain knockout mice. During the proliferative expansion, expression of the genes encoding SL chain is downregulated and the SL chain protein disappears from the cell. When pre-B cells leave the cell cycle and become small pre-B cells, light chain genes are rearranged that encode for a light chain protein forming a B cell receptor with the selected μH chain. Five cell divisions at the pre-B cell stage are depicted, leading to 32-fold higher output of B cells in wild type vs. SL knockout mice. See text for further details.

The heavily cycling status of pre-BCR positive cells and the detection of a significant population of cμHC-positive CD25^+^ pre-B cells that apparently have downregulated SL chain expression [(42–44, 46)] before they become small pre-B cells led the group of Rolink and Melchers to propose the model of proliferative expansion of pre-B cells as a major function of the SL chain (49). According to this model that is now widely accepted, the pre-BCR induces the proliferative expansion of pre-B cells that have undergone successful V_H_DJ_H_ recombination and express a μHC (49) (Figure 2). This also explained the observation that Rag-deficient mice expressing a μH chain transgene fill up the pre-B cell compartment to normal numbers (42). In addition, this model explains the phenotype of the λ5 knockout mouse (Figure 2). In the absence of proliferative expansion pre-B cells could develop further albeit with very low efficiency to the small pre-B cell stage in which light chain recombination is activated. Interestingly, Decker et al. proposed already in 1991 that pre-B cells would undergo five to six divisions subsequent to V_H_ to DJ_H_ rearrangement (50). A publication by Rolink et al. showed that even in culture medium sorted pro-B become pre-B cells *in vitro* and divide spontaneously up to six times (51). This cell division was not observed, when pro-B cells from λ5 knockout mice were sorted and cultured. Finally, Hess et al. directly demonstrated induction of proliferation mediated by the pre-BCR using a tetracyclin-system where μH chain expression could be switched on and off in Rag-deficient pro-B cells (52). Using five to six cell divisions as an assumption for cells at the pre-B stage, λ5T mice would have a 32- to 64-fold reduced pre-B cell compartment and accordingly a similar decrease in immature B cells. This reflects almost exactly the reduction of the pre-B and immature B cell compartment originally suggested (20) and also obtained by the quantitative analysis of the λ5 knockout mice published by Rolink et al. (53). The SL chain is necessary to generate large enough numbers of B cells (Figure 2). Although the proliferative expansion of pre-B cells generates cells with identical IgH rearrangements this is not a problem for repertoire diversity, as each cell will randomly rearrange and express a different IgL chain (Figure 2). In addition, and discussed later, the pre-BCR considerably shapes the V_H_-repertoire.

## The role of the pre-B cell receptor for allelic exclusion

The ordered rearrangement model of B cell development also supports a regulated mechanism of IgH allelic exclusion in which a V_H_DJ_H_ rearrangement, if productive, prevents an additional V_H_-to-DJ_H_ rearrangement on the other allele (34). As this feedback inhibition model predicted, the targeted disruption of μHC membrane exon was shown to cause loss of IgH allelic exclusion (54). Surprisingly, in the λ5T mouse allelic exclusion was found to be perfectly normal, when analyzing mature B cells (20). This was puzzling and suggested to the authors that *bona fide* IgL chains could substitute for SL chain. Whereas, it was consistently found that IgL recombination does not require expression of SL chain expression or μH chain (36, 37), it was vividly discussed at the International Titisee Conference in 1994^1^, whether IgL rearrangement is occurring at early stages of B cell development (36, 44, 55, 56), and hence could substitute for SL chain in the λ5T mouse, including signaling of allelic exclusion.

It was surprising when the new technology of single-cell rearrangement PCR revealed that μH chain double-producing B precursor cells are generated in λ5T mice but apparently not in wild type mice (57). These μHC double-producing cells do not appear as surface μH chain double-positive cells, however (57). The most likely explanation for this conundrum was provided by the finding that pre-B cells showing allelic inclusion display allelic exclusion at the level of pre-BCR surface expression (58). This study found that double-producers were present also in wild type mice. In cells with both alleles functionally rearranged, i.e., allelic inclusion, only one of the μHCs is able to be expressed the cell surface. Similar findings were published for a μHC that was unable to pair with the SL but also with *bona fide* IgL chains (59). It was therefore proposed that one important function of the SL chain is to test for pairing capabilities of newly formed μHCs for later stages of development when the *bona fide* LC is rearranged (60). In light of the apparently conflicting results regarding allelic inclusion at the level of VDJ-rearrangement, this concept offers an explanation that currently is generally agreed upon (61).

As a potential mechanism for an immediate signaling of feedback inhibition, the complete downregulation of Rag-1 and Rag-2 transcription in pre-BCR positive pre-B cells was described (62). In addition, the Rag-2 protein was found to be destabilized in the S phase of these rapidly dividing cells (63, 62). Additional mechanisms at the chromatin level have to be operating to assure allelic exclusion at later stages of development, when the Rag-genes are re-expressed, at the small pre-B cell stage [reviewed in (64, 65)]. It is interesting, however, that μH chains can reach the cell surface and still have the capacity to signal in the absence of SL chain, including down-regulation of the V(D)J-recombinase machinery (21, 66, 67).

## Humoral immunodeficiency in patients with mutated SL genes

In humans, the SL chains and the pre-BCR are expressed at corresponding stages (48, 68, 69). In 1998, the detailed molecular analysis of 8 patients with sporadic agammaglobulinemia for mutations in candidate genes revealed one patient with mutations on both alleles of the gene for λ5 (70). In this patient, B cell development shows a complete block of differentiation as B cells were undetectable up to an age of 5 years. Whereas, the maternal allele carried a stop codon in the first exon of λ5 the paternal allele demonstrated a three-base pair substitution in exon 3. A similar, broader sequencing analysis of 33 patients with primary immunodeficiency discovered two sisters being homozygous for a deletion in the λ5 gene in exon 2 (71). As no additional clinical details were provided, it remains unclear whether also in these two sisters B lymphopoiesis is completely blocked. It is still unknown, why the phenotype of mutations in the SL chain in humans have a much more complete phenotype regarding B cell development. Interestingly, mutations in Bruton's tyrosine kinase *btk* leads to an almost complete block in B cell development in humans but only a mild block in btk-deficient mice (72). This suggests different signaling requirements for mouse and human pre-B cell development, as illustrated by apparently different levels of redundancy of Tec kinase members in humans and mice (73).

## Cell autonomous signaling and/or ligand-mediated signaling by the pre-BCR

Mouse pro-B cells that do not express a pre-BCR (μH^−^) require stromal cells and high levels of IL-7 in order to proliferate. This is in contrast to pre-B cells that do not require stromal cells but do require a pre-BCR and low concentrations of IL-7 (51, 66, 74–76). The latter was interpreted as ligand-independent cell autonomous signaling. Pre-BCR-mediated signaling in a cell autonomous manner has been confirmed, and shown to rely on a particular aa residue in the μH chain (N46) (77), although whether this signal takes place between receptors on the one and same cell, on neighboring cells or both is unclear. Nevertheless, this does not exclude that the pre-BCR can also interact with a ligand. Indeed, at least two ligands have been described. Early work demonstrated the importance of Galectin-1, produced by stromal cells, as a pre-BCR ligand in humans, which requires the λ5-UR (78). In mice, stromal cell associated heparan sulfate was shown to be important, which would engage with the λ5-UR in the context of a pre-BCR (79). A potential explanation for different ligands could be that there are differences between mouse and human (79), that the stromal cells that produce IL7 are not the same as those that produce galectin-1, and are located in different BM niches (80). As the pre-BCR mediates several signals this may also account for different requirements. Nevertheless, whether the pre-BCR mediates signals in a ligand-dependent or -independent manner, cell surface levels depend on the respective UR. The λ5-UR is required for rapid internalization and signaling; in its absence mutant receptors with reduced signalling capacity accumulate on the surface (81). By contrast, the absence of the VpreB1-UR increases internalisation and hence was concluded to balance the rate of internalization (47). Moreover, pre-BCR surface levels are important as they seemingly regulate both proliferation and survival (82).

## The pre-BCR shapes the IgH repertoire

In mice, there are 195 V_H_ gene segments of which 110 are functional and can be divided into 16 families (83). Among the V_H_ genes the V_H_1 (J558), V_H_2 (Q52), and V_H_5 (7183) are the most studied, for several reasons. For instance, usage of the DJ_H_-proximal V_H_ genes, V_H_2 and V_H_5, and especially V_H_5-2 (81X), is especially high in the fetal and neonatal repertoire (84, 85). Differentiating B cells of adult bone marrow mimic fetal development and in adult BM V_H_ usage changes at the pro-B to pre-B cell transition (86, 87) whereas it is not markedly changed at later stages. At the pre-B cell stage V_H_1 usage increases whilst that of V_H_5 decreases. The discovery of the pre-BCR and that it is expressed at a time during BM B cell development when the V_H_ repertoire changes indicated its potential involvement. This was investigated early on in λ5T mice. The results confirmed previous studies in wild type mice, which is quite remarkable considering that the studies were performed by single cell PCR analyzing 25–30 cells per population (88). The low numbers were due to low detection levels of both alleles in each cell, and the number of cellular fractions analyzed, hence a remarkable accomplishment almost 25 years ago. At that time, pre-B cells expressing intracellular μH chains and splenic follicular B cells from λ5T mice were analyzed, which showed a more frequent usage of the V_H_5 and V_H_2 (Q52) genes and less frequent usage of V_H_1 genes among pre-B cells. Therefore, the preBCR contributes to repertoire selection at the preB cell stage. However, V_H_ usage in the spleen of λ5T mice was similar to the corresponding cells from wild type control mice. The interpretation was that when there was no SL chain, the change in the repertoire observed in splenic B cells must be mediated by *bona fide* light chains, although at a later stage in development.

In light of the close interactions of the VpreB chain with the IgH chain complementarity region 3 (H-CDR3) in the crystal structure of the preBCR (16) an influence of the preBCR on the shaping of the antibody repertoire was postulated. Detailed analysis indeed revealed that a particular amino acid composition in the H-CDR3, in particular tyrosine at position 101 is positively selected by interactions with VpreB (89). Interestingly, in the mouse the combination of evolutionary selection of a preferred reading frame usage in the D-elements as well as somatic selection by the invariant SL chain together favour the presence of tyrosine at key positions in the antigen-binding site of antibodies (89). As this tyrosine residue is frequently found in contact with the antigen in antibody/antigen complexes, preBCR selection directly influences the antigen binding characteristics of the mature antibody repertoire (90).

## A role for the pre-BCR in B cell tolerance?

Immature B cells expressing poly- and/or autoreactive BCRs undergo negative selection in order to prevent their development into naïve B cells. It has been suggested, however, that at the pre-B stage poly-reactivity is a requirement for positive selection and expansion in mice (91). The poly-/autoreactivity relates to the λ5-UR with its high number of positively charged aa residues. These are mainly arginine, an aa typically found in anti-DNA and polyreactive antibodies, e.g., in the (H-CDR3) (92). As mentioned above, pre-BCR signalling is reduced in the absence of the λ5-UR. However, the λ5-UR can be replaced by a polyreactive H-CDR3 (from human antibodies) resulting in a signalling competent receptor (91). This lead to the conclusion that the pre-BCR is “self-reactive,” and that the “autoreactivity” is driven by the λ5-UR and is required for positive selection of pre-B cells.

The pre-BCR appears to also counter-selects particular μH chains, in fact those that express a H-CDR3 with basic aa residues, e.g., arginine (93, 94). In wild type control mice, this selection takes place in pro-B cells, i.e., at the transition into the pre-B cell stage and was based on the analyses of the V_H_5 family (94), a selection that is not as evident when analyzing all V_H_ genes by NGS (95). Nevertheless, the absence of the entire SL chain (SLC^−/−^) in mice results in pre-B cells expressing μH chains with a much higher proportion of basic aa residues in the H-CDR3 (95). Whether this is a result of positive selection and proliferative expansion, in line with the above-mentioned requirement for poly-/autoreactivity is currently unclear (91), as at least some of the expansion is likely due to signaling through the IL-7R (66, 76). Anyhow, negative selection at the immature B cell stage in SLC^−/−^ mice is more prominent than in controls, inferred from a higher proportion of cells prone to apoptosis (96), and interpreted as a result of the expansion of “autoreactive” pre-B cells (95). Central B-cell tolerance is despite of this incomplete. In addition, also peripheral B-cell tolerance is incomplete, and results in a higher proportion of splenic FO B cells expressing μH chains with basic aa residues in their H-CDR3 (95). A subset of the FO B cells are activated and initiates autoimmune reactions. Whether this is due to SL chain being required for termination of signaling earlier in development is currently unclear (97). Nevertheless, the autoimmune reactions include spontaneous formation of T-cell dependent germinal centers, memory B cells and plasma cells that secrete autoantibodies, typical of those found in lupus (SLE), e.g., anti-DNA and anti-nuclear antibodies (ANAs) (94, 95). Whether this is unique to mice lacking the entire SL chain is unclear, although splenic B cells in λ5T mice are also enriched for those expressing IgH chains with an arginine-rich H-CDR3 (98), and more recent work has shown that also λ5T mice secrete autoantibodies (90). In SLC^−/−^ mice a subset of B cells resembles memory B cells expressing low levels of CD21 (CD21^−/low^) (99). Memory B cells with a CD21^−/low^ phenotype expand under conditions of chronic immune stimulation in humans, e.g., in patients with autoimmune disease, SLE and RA, or pathogenic infections, malaria, and HIV (100). CD21^−/low^ B cells have also been described in wild type control mice, termed age associated B cells (ABCs) as they accumulate with age (101). However, at least at a young age most of the ABCs in wild type control mice are not memory B cells whereas those in SLC^−/−^ mice are (99). Moreover, the ABCs in SLC^−/−^ mice are not polyreactive but rather autoreactive producing typical lupus autoantibodies, e.g., anti-Smith antigens. The ABCs are mainly IgM that show signs of somatic hypermutation, and strong selection of the H-CDR3, whereas the GC B cells are mainly IgG2c^+^ and likely the source of the plasma cells that produce the serum anti-DNA and ANAs (94, 95). Perhaps surprisingly, the small number of ANA-reactive ABC hybridomas analyzed so far did not show any signs of somatic hypermutations in the V_H_. However, whether they express IgL chains with mutations is currently unclear. In this context it was recently shown only that some of the ANA-reactive hybridomas from aged mice also express germ line encoded IgH chains, and that the autoreactivity was due to mutations in the IgL chain (102). In fact, substitution of one aa residue in the Igk CDR1 was sufficient to convert the antibody to being ANA-reactive. The similarities between the hybridomas from aged mice and the ABCs in SLC^−/−^ mice could be taken as an indication that aged mice are reminiscent of young SLC^−/−^ mice. However, whether this is the case requires additional studies.

## Conclusion

We conclude that pre-BCR mediated signaling has been implicated in:

- Proliferation
- Survival
- Downregulation of the RAG recombinases
- IgH allelic exclusion
- Silencing of the genes encoding SL chain
- Selection of the IgH repertoire
- Positive selection of pre-B cells
- Negative selection

It should be noted though that we still do not fully understand all these events, and especially not which signals are mediated at what stage. For instance, it has been proposed that the pre-BCR mediates early and late signals (103). The early signals might be those taking place in the pre-BCR^+^ pro-B (pre-B1) cells (c-kit^+^CD25^−^) and the late signals in the pre-BCR^+^ pre-B (large pre-BII) cells (c-kit^−^CD25^+^). In addition, the role of the SL chain in human pre-B cell development is only partially understood. New technologies as CRISPR/Cas9 gene editing and humanized mice would now allow the analysis of this question.

## Author contributions

All authors listed have made a substantial, direct and intellectual contribution to the work, and approved it for publication.

### Conflict of interest statement

The authors declare that the research was conducted in the absence of any commercial or financial relationships that could be construed as a potential conflict of interest.

## Footnotes

^1^https://www.bifonds.de/titisee-conferences/past-conferences/past-conference/items/titisee-conferences-pastitc_19941005-70itc.html

## References

[1] Burrows P LeJeune M Kearney JF. Evidence that murine pre-B cells synthesise μ heavy chains but no light chains. Nature (1979) 280:838–40. 10.1038/280838a0 112480

[2] Siden EJ Baltimore D Clark D Rosenberg NE. Immunoglobulin synthesis by lymphoid cells transformed *in vitro* by Abelson murine leukemia virus. Cell (1979) 16:389–96. 10.1016/0092-8674(79)90014-X 222460

[3] Sakaguchi N Melchers F. Lambda 5, a new light-chain-related locus selectively expressed in pre-B lymphocytes. Nature (1986) 324:579–82. 10.1038/324579a0 3024017

[4] Pillai S Baltimore D. Formation of disulphide-linked mu 2 omega 2 tetramers in pre-B cells by the 18K omega-immunoglobulin light chain. Nature (1987) 329:172–4. 10.1038/329172a0 3114643

[5] Kudo A Sakaguchi N Melchers F. Organization of the murine Ig-related lambda 5 gene transcribed selectively in pre-B lymphocytes. Embo J. (1987) 6:103–7. 10.1002/j.1460-2075.1987.tb04725.x 3107979 PMC553363

[6] Kudo A Melchers F. A second gene, VpreB in the lambda 5 locus of the mouse, which appears to be selectively expressed in pre-B lymphocytes. Embo J. (1987) 6:2267–72. 10.1002/j.1460-2075.1987.tb02500.x 3117530 PMC553628

[7] Dul JL Argon Y Winkler T ten Boekel E Melchers F Martensson IL. The murine VpreB1 and VpreB2 genes both encode a protein of the surrogate light chain and are co-expressed during B cell development. Eur J Immunol. (1996) 26:906–13. 10.1002/eji.1830260428 8625987

[8] Bauer SR Kudo A Melchers F. Structure and pre-B lymphocyte restricted expression of the VpreB in humans and conservation of its structure in other mammalian species. EMBO J. (1988) 7:111–6. 10.1002/j.1460-2075.1988.tb02789.x 3258819 PMC454222

[9] Chang H Dmitrovsky E Hieter PA Mitchell K Leder P Turoczi L. Identification of three new Ig lambda-like genes in man. J Exp Med. (1986) 163:425–35. 10.1084/jem.163.2.425 3003227 PMC2188038

[10] Hollis GF Evans RJ Stafford-Hollis JM Korsmeyer SJ McKearn JP. Immunoglobulin lambda light-chain-related genes 14.1 and 16.1 are expressed in pre-B cells and may encode the human immunoglobulin omega light-chain protein. Proc Natl Acad Sci USA. (1989) 86:5552–6. 10.1073/pnas.86.14.5552 2501791 PMC297661

[11] Conley ME Rapalus L Boylin EC Rohrer J Minegishi Y. Gene conversion events contribute to the polymorphic variation of the surrogate light chain gene lambda 5/14.1. Clin Immunol. (1999) 93:162–7. 10.1006/clim.1999.4785 10527692

[12] Karasuyama H Kudo A Melchers F. The proteins encoded by the VpreB and lambda 5 pre-B cell-specific genes can associate with each other and with mu heavy chain. J Exp Med. (1990) 172:969–72. 10.1084/jem.172.3.969 2117638 PMC2188555

[13] Tsubata T Reth M. The products of pre-B cell-specific genes (lambda 5 and VpreB) and the immunoglobulin mu chain form a complex that is transported onto the cell surface. J Exp Med. (1990) 172:973–6. 10.1084/jem.172.3.973 2117639 PMC2188549

[14] Minegishi Y Hendershot LM Conley ME. Novel mechanisms control the folding and assembly of lambda5/14.1 and VpreB to produce an intact surrogate light chain. Proc Natl Acad Sci USA. (1999) 96:3041–6. 10.1073/pnas.96.6.3041 10077633 PMC15891

[15] Lanig H Bradl H Jack HM. Three-dimensional modeling of a pre-B-cell receptor. Mol Immunol. (2004) 40:1263–72. 10.1016/j.molimm.2003.11.030 15128043

[16] Bankovich AJ Raunser S Juo ZS Walz T Davis MM Garcia KC. Structural insight into pre-B cell receptor function. Science (2007) 316:291–4. 10.1126/science.1139412 17431183

[17] Elantak L Espeli M Boned A Bornet O Bonzi J Gauthier L. Structural basis for galectin-1-dependent pre-B cell receptor (pre-BCR) activation. J Biol Chem. (2012) 287:44703–13. 10.1074/jbc.M112.395152 23124203 PMC3531785

[18] Thomas KR Capecchi MR. Site-directed mutagenesis by gene targeting in mouse embryo-derived stem cells. Cell (1987) 51:503–12. 10.1016/0092-8674(87)90646-5 2822260

[19] Thompson S Clarke AR Pow AM Hooper ML Melton DW. Germ line transmission and expression of a corrected HPRT gene produced by gene targeting in embryonic stem cells. Cell (1989) 56:313–21. 10.1016/0092-8674(89)90905-7 2912572

[20] Kitamura D Kudo A Schaal S Müller W Melchers F Rajewsky K. A critical role of lambda 5 protein in B cell development. Cell (1992) 69:823–31. 10.1016/0092-8674(92)90293-L 1591779

[21] Mundt C Licence S Shimizu T Melchers F Mårtensson IL Loss of precursor B cell expansion but not allelic exclusion in VpreB1/VpreB2 double-deficient mice. J Exp Med. (2001) 193:435–45. 10.1084/jem.193.4.435 11181696 PMC2195903

[22] Mårtensson A Argon Y Melchers F Dul JL Mårtensson IL. Partial block in B lymphocyte development at the transition into the pre-B cell receptor stage in Vpre-B1-deficient mice. Int Immunol. (1999) 11:453–60. 10.1093/intimm/11.3.453 10221657

[23] Mundt C Licence S Maxwell G Melchers F Martensson IL Only VpreB1, but not VpreB2, is expressed at levels which allow normal development of B cells. Int Immunol. (2006) 18:163–72. 10.1093/intimm/dxh359 16361315

[24] Shimizu T Mundt C Licence S Melchers F Mårtensson I-L. VpreB1/VpreB2/lambda 5 triple-deficient mice show impaired B cell development but functional allelic exclusion of the IgH locus. J Immunol. (2002) 168:6286–93. 10.4049/jimmunol.168.12.6286 12055243

[25] Kitamura D Roes J Kühn R Rajewsky K. A B cell-deficient mouse by targeted disruption of the membrane exon of the immunoglobulin mu chain gene. Nature (1991) 350:423–6. 10.1038/350423a0 1901381

[26] Mombaerts P Iacomini J Johnson RS Herrup K Tonegawa S Papaioannou VE. RAG-1-deficient mice have no mature B and T lymphocytes. Cell (1992) 68:869–77. 10.1016/0092-8674(92)90030-G 1547488

[27] Shinkai Y Rathbun G Lam KP Oltz EM Stewart V Mendelsohn M. RAG-2-deficient mice lack mature lymphocytes owing to inability to initiate V(D)J rearrangement. Cell (1992) 68:855–67. 10.1016/0092-8674(92)90029-C 1547487

[28] Reth M Hombach J Wienands J Campbell KS Chien N Justement LB. The B-cell antigen receptor complex. Immunol Today (1991) 12:196–201. 10.1016/0167-5699(91)90053-V 1878135

[29] Venkitaraman AR Williams GT Dariavach P Neuberger MS. The B-cell antigen receptor of the five immunoglobulin classes. Nature (1991) 352:777–81. 10.1038/352777a0 1881434

[30] Gong S Nussenzweig MC. Regulation of an early developmental checkpoint in the B cell pathway by Ig beta. Science (1996) 272:411–4. 10.1126/science.272.5260.411 8602530

[31] Pelanda R Braun U Hobeika E Nussenzweig MC Reth M B cell progenitors are arrested in maturation but have intact VDJ recombination in the absence of Ig-a and Ig-b. J Immunol. (2002) 169:865–72. 10.4049/jimmunol.169.2.865 12097390

[32] Bosma GC Fried M Custer RP Carroll A Gibson DM Bosma MJ. Evidence of functional lymphocytes in some (leaky) scid mice. J Exp Med. (1988) 167:1016–33. 10.1084/jem.167.3.1016 3280724 PMC2188881

[33] Fehling HJ Krotkova A Saint-Ruf C von Boehmer H Crucial role of the pre-T-cell receptor alpha gene in development of alpha beta but not gamma delta T cells. Nature (1995) 375:795–8. 10.1038/375795a0 7596413

[34] Alt FW Yancopoulos GD Blackwell TK Wood C Thomas E Boss M. Ordered rearrangement of immunoglobulin heavy chain variable region segments. EMBO J. (1984) 3:1209–19. 10.1002/j.1460-2075.1984.tb01955.x 6086308 PMC557501

[35] Reth MG Ammirati P Jackson S Alt FW. Regulated progression of a cultured pre-B-cell line to the B-cell stage. Nature (1985) 317:353–5. 10.1038/317353a0 3930970

[36] Ehlich A Schaal S Gu H Kitamura D Müller W Rajewsky K. Immunoglobulin heavy and light chain genes rearrange independently at early stages of B cell development. Cell (1993) 72:695–704. 10.1016/0092-8674(93)90398-A 8453664

[37] Grawunder U Haasner D Melchers F Rolink A. Rearrangement and expression of kappa light chain genes can occur without mu heavy chain expression during differentiation of pre-B cells. Int Immunol. (1993) 5:1609–18. 10.1093/intimm/5.12.1609 8312230

[38] Hardy RR Carmack CE Shinton SA Kemp JD Hayakawa K. Resolution and characterization of pro-B and pre-pro-B cell stages in normal mouse bone marrow. J Exp Med. (1991) 173:1213–25. 10.1084/jem.173.5.1213 1827140 PMC2118850

[39] Osmond DG. Proliferation kinetics and the lifespan of B cells in central and peripheral lymphoid organs. Curr Opin Immunol. (1991) 3:179–85. 10.1016/0952-7915(91)90047-5 2069745

[40] Rolink A Melchers F. Molecular and cellular origins of B lymphocyte diversity. Cell (1991) 66:1081–94. 10.1016/0092-8674(91)90032-T 1913803

[41] Rolink A Streb M Nishikawa S Melchers F. The c-kit-encoded tyrosine kinase regulates the proliferation of early pre-B cells. Eur J Immunol. (1991b) 21:2609–12. 10.1002/eji.1830211044 1717287

[42] Rolink A Grawunder U Winkler TH Karasuyama H Melchers F. IL-2 receptor alpha chain (CD25, TAC) expression defines a crucial stage in pre-B cell development. Int Immunol. (1994) 6:1257–64. 10.1093/intimm/6.8.1257 7526894

[43] Karasuyama H Rolink A Shinkai Y Young F Alt FW Melchers F. The expression of Vpre-B/lambda 5 surrogate light chain in early bone marrow precursor B cells of normal and B cell-deficient mutant mice. Cell (1994) 77:133–43. 10.1016/0092-8674(94)90241-0 8156589

[44] Li YS Hayakawa K Hardy RR. The regulated expression of B lineage associated genes during B cell differentiation in bone marrow and fetal liver. J Exp Med. (1993) 178:951–60. 10.1084/jem.178.3.951 8350062 PMC2191150

[45] Lassoued K Nuñez CA Billips L Kubagawa H Monteiro RC LeBlen TW. Expression of surrogate light chain receptors is restricted to a late stage in pre-B cell differentiation. Cell (1993) 73:73–86. 10.1016/0092-8674(93)90161-I 7681728

[46] Winkler TH Rolink AG Melchers F Karasuyama H. Precursor B cells of mouse bone marrow express two different complexes with the surrogate light chain on the surface. J Immunol. (1995) 25:446–50. 7875207 10.1002/eji.1830250221

[47] Knoll M Yanagisawa Y Simmons S Engels N Wienands J Melchers F. The non-Ig parts of the VpreB and lambda5 proteins of the surrogate light chain play opposite roles in the surface representation of the precursor B cell receptor. J Immunol. (2012) 188:6010–7. 10.4049/jimmunol.1200071 22566564

[48] Meffre E Fougereau M Argenson JN Aubaniac JM Schiff C Cell surface expression of surrogate light chain (YL) in the absence of m on human pro-B cell lines and normal pro-B cells. Eur J Immunol. (1996) 26:2172–80. 10.1002/eji.1830260932 8814264

[49] Melchers F Rolink A Grawunder U Winkler TH Karasuyama H Ghia P. Positive and negative selection events during B lymphopoiesis. Curr Opin Immunol. (1995) 7:214–27. 10.1016/0952-7915(95)80006-9 7546381

[50] Decker DJ Boyle NE Koziol JA Klinman NR. The expression of the Ig H chain repertoire in developing bone marrow B lineage cells. J Immunol. (1991) 146:350–61. 1898607

[51] Rolink AG Winkler T Melchers F Andersson J Precursor B cell receptor-dependent B cell proliferation and differentiation does not require the bone marrow or fetal liver environment. J Exp Med. (2000) 191:23–32. 10.1084/jem.191.1.23 10620602 PMC2195801

[52] Hess J Werner A Wirth T Melchers F Jäck HM Winkler TH. Induction of pre-B cell proliferation after *>de novo* synthesis of the pre-B cell receptor. Proc Natl Acad Sci USA. (2001) 98:1745–50. 10.1073/pnas.98.4.1745 11172022 PMC29328

[53] Rolink A Karasuyama H Grawunder U Haasner D Kudo A Melchers F. B cell development in mice with a defective lambda 5 gene. Eur J Immunol. (1993) 23:1284–8. 10.1002/eji.1830230614 7684685

[54] Kitamura D Rajewsky K. Targeted disruption of mu chain membrane exon causes loss of heavy-chain allelic exclusion. Nature (1992) 356:154–6. 10.1038/356154a0 1545868

[55] ten Boekel E Melchers F Rolink A The status of Ig loci rearrangements in single cells from different stages of B cell development. Int Immunol. (1995) 7:1013–9. 10.1093/intimm/7.6.1013 7577795

[56] Pelanda R Schaal S Torres RM Rajewsky K A prematurely expressed Ig(kappa) transgene, but not V(kappa)J(kappa) gene segment targeted into the Ig(kappa) locus, can rescue B cell development in lambda5-deficient mice. Immunity (1996) 5:229–39. 10.1016/S1074-7613(00)80318-0 8808678

[57] Löffert D Ehlich A Müller W Rajewsky K. Surrogate light chain expression is required to establish immunoglobulin heavy chain allelic exclusion during early B cell development. Immunity (1996) 4:133–44. 10.1016/S1074-7613(00)80678-0 8624804

[58] ten Boekel E Melchers F Rolink AG Precursor B cells showing H chain allelic inclusion display allelic exclusion at the level of pre-B cell receptor surface expression. Immunity (1998) 8:199–207. 10.1016/S1074-7613(00)80472-0 9492001

[59] Kline GH Hartwell L Beck-Engeser GB Keyna U Zaharevitz S Klinman NR. Pre-B cell receptor-mediated selection of pre-B cells synthesizing functional mu heavy chains. J Immunol. (1998) 161:1608–18. 9712022

[60] Vettermann C Herrmann K Jäck HM. Powered by pairing: the surrogate light chain amplifies immunoglobulin heavy chain signaling and pre-selects the antibody repertoire. Semin Immunol. (2006) 18:44–55. 10.1016/j.smim.2006.01.001 16464608

[61] Melchers F. The pre-B-cell receptor: selector of fitting immunoglobulin heavy chains for the B-cell repertoire. Nat Rev Immunol. (2005) 5:578–84. 10.1038/nri1649 15999097

[62] Grawunder U Leu TM Schatz DG Werner A Rolink AG Melchers F. Down-regulation of RAG1 and RAG2 gene expression in preB cells after functional immunoglobulin heavy chain rearrangement. Immunity (1995) 3:601–8. 10.1016/1074-7613(95)90131-0 7584150

[63] Lin WC Desiderio S. Cell cycle regulation of V(D)J recombination-activating protein RAG-2. Proc Natl Acad Sci USA. (1994) 91:2733–7. 10.1073/pnas.91.7.2733 8146183 PMC43444

[64] Vettermann C Schlissel MS. Allelic exclusion of immunoglobulin genes: models and mechanisms. Immunol Rev. (2010) 237:22–42. 10.1111/j.1600-065X.2010.00935.x 20727027 PMC2928156

[65] Levin-Klein R Bergman Y. Epigenetic regulation of monoallelic rearrangement (allelic exclusion) of antigen receptor genes. Front Immunol. (2014) 5:625. 10.3389/fimmu.2014.00625 25538709 PMC4257082

[66] Schuh W Meister S Roth E Jäck HM. Cutting edge: signaling and cell surface expression of a mu H chain in the absence of lambda 5: a paradigm revisited. J Immunol. (2003) 171:3343–7. 10.4049/jimmunol.171.7.3343 14500626

[67] Galler GR Mundt C Parker M Pelanda R Mårtensson IL Winkler TH. Surface mu heavy chain signals down-regulation of the V(D)J-recombinase machinery in the absence of surrogate light chain components. J Exp Med. (2004) 199:1523–32. 10.1084/jem.20031523 15173209 PMC2211789

[68] Ghia P A ten Boekel E Sanz E de la Hera A Rolink A Melchers F. Ordering of human bone marrow B lymphocyte precursors by single-cell polymerase chain reaction analyses of the rearrangement status of the immunoglobulin H and L chain gene loci. J Exp Med. (1996) 184:2217–29. 10.1084/jem.184.6.2217 8976177 PMC2196361

[69] Wang YH Nomura J Faye-Petersen OM Cooper MD. Surrogate light chain production during B cell differentiation: differential intracellular versus cell surface expression. J Immunol. (1998) 161:1132–9. 9686571

[70] Minegishi Y Coustan-Smith E Wang YH Cooper MD Campana D Conley ME. Mutations in the human lambda5/14.1 gene result in B cell deficiency and agammaglobulinemia. J Exp Med. (1998) 187:71–7. 10.1084/jem.187.1.71 9419212 PMC2199185

[71] Moens LN Falk-Sorqvist E Asplund AC Bernatowska E Smith CI Nilsson M. Diagnostics of primary immunodeficiency diseases: a sequencing capture approach. PLoS ONE (2014) 9:e114901. 10.1371/journal.pone.0114901 25502423 PMC4263707

[72] Hendriks RW de Bruijn MF Maas A Dingjan GM Karis A Grosveld F. Inactivation of Btk by insertion of lacZ reveals defects in B cell development only past the pre-B cell stage. EMBO J. (1996) 15:4862–72. 10.1002/j.1460-2075.1996.tb00867.x 8890160 PMC452224

[73] Ellmeier W Jung S Sunshine MJ Hatam F Xu Y Baltimore D. Severe B cell deficiency in mice lacking the tec kinase family members Tec and Btk. J Exp Med. (2000) 192:1611–24. 10.1084/jem.192.11.1611 11104803 PMC2193106

[74] Rolink A Kudo A Karasuyama H Kikuchi Y Melchers F. Long-term proliferating early pre B cell lines and clones with the potential to develop to surface Ig-positive, mitogen reactive B cells *in vitro* and *in vivo*. Embo J. (1991a) 10:327–36. 10.1002/j.1460-2075.1991.tb07953.x 1991449 PMC452650

[75] Ray RJ Stoddart A Pennycook JL Huner HO Furlonger C Wu GE. Stromal cell-independent maturation of IL-7-responsive pro-B cells. J Immunol. (1998) 160:5886–97. 9637501

[76] Erlandsson L Licence S Gaspal F Lane P Corcoran AE Martensson IL. Both the pre-BCR and the IL-7Ralpha are essential for expansion at the pre-BII cell stage *in vivo*. Eur J Immunol. (2005) 35:1969–76. 10.1002/eji.200425821 15909309

[77] Ubelhart R Bach MP Eschbach C Wossning T Reth M Jumaa H. N-linked glycosylation selectively regulates autonomous precursor BCR function. Nat Immunol. (2010) 11:759–65. 10.1038/ni.1903 20622883

[78] Gauthier L Rossi B Roux F Termine E Schiff C. Galectin-1 is a stromal cell ligand of the pre-B cell receptor (BCR) implicated in synapse formation between pre-B and stromal cells and in pre-BCR triggering. Proc Natl Acad Sci USA. (2002) 99:13014–9. 10.1073/pnas.202323999 12271131 PMC130578

[79] Bradl H Wittmann J Milius D Vettermann C Jack HM. Interaction of murine precursor B cell receptor with stroma cells is controlled by the unique tail of lambda 5 and stroma cell-associated heparan sulfate. J Immunol. (2003) 171:2338–48. 10.4049/jimmunol.171.5.2338 12928380

[80] Mourcin F Breton C Tellier J Narang P Chasson L Jorquera A. Galectin-1-expressing stromal cells constitute a specific niche for pre-BII cell development in mouse bone marrow. Blood (2011) 117:6552–61. 10.1182/blood-2010-12-323113 21511956

[81] Ohnishi K Melchers F. The nonimmunoglobulin portion of lambda5 mediates cell-autonomous pre-B cell receptor signaling. Nat Immunol. (2003) 4:849–56. 10.1038/ni959 12897780

[82] Kawano Y Yoshikawa S Minegishi Y Karasuyama H. Pre-B cell receptor assesses the quality of IgH chains and tunes the pre-B cell repertoire by delivering differential signals. J Immunol. (2006) 177:2242–9. 10.4049/jimmunol.177.4.2242 16887984

[83] Johnston CM Wood AL Bolland DJ Corcoran AE. Complete sequence assembly and characterization of the C57BL/6 mouse Ig heavy chain V region. J Immunol. (2006) 176:4221–34. 10.4049/jimmunol.176.7.4221 16547259

[84] Wu GE Paige CJ. VH gene family utilization in colonies derived from B and pre-B cells detected by the RNA colony blot assay. Embo J. (1986) 5:3475–81. 10.1002/j.1460-2075.1986.tb04672.x 2435544 PMC1167383

[85] Yancopoulos GD Malynn BA Alt FW. Developmentally regulated and strain-specific expression of murine VH gene families. J Exp Med. (1988) 168:417–35. 10.1084/jem.168.1.417 3135366 PMC2188955

[86] Freitas AA Andrade L Lembezat MP Coutinho A. Selection of VH gene repertoires: differentiating B cells of adult bone marrow mimic fetal development. Int Immunol. (1990) 2:15–23. 10.1093/intimm/2.1.15 2128463

[87] Huetz F Carlsson L Tornberg UC Holmberg D. V-region directed selection in differentiating B lymphocytes. EMBO J. (1993) 12:1819–26. 10.1002/j.1460-2075.1993.tb05830.x 8491175 PMC413401

[88] ten Boekel E Melchers F Rolink AG Changes in the V(H) gene repertoire of developing precursor B lymphocytes in mouse bone marrow mediated by the pre-B cell receptor. Immunity (1997) 7:357–68. 10.1016/S1074-7613(00)80357-X 9324356

[89] Khass M Blackburn T Burrows PD Walter MR Capriotti E Schroeder HW Jr VpreB serves as an invariant surrogate antigen for selecting immunoglobulin antigen-binding sites. Sci Immunol. (2016) 1:aaf6628. 10.1126/sciimmunol.aaf6628 PMC5315267 28217764

[90] Khass M Blackburn T Elgavish A Burrows PD Schroeder HWJr. In the absence of central pre-B cell receptor selection, peripheral selection attempts to optimize the antibody repertoire by enriching for CDR-H3 Y101. Front Immunol. (2018) 9:120. 10.3389/fimmu.2018.00120 29472919 PMC5810287

[91] Kohler F Hug E Eschbach C Meixlsperger S Hobeika E Kofer J. Autoreactive B cell receptors mimic autonomous pre-B cell receptor signaling and induce proliferation of early B cells. Immunity (2008) 29:912–21. 10.1016/j.immuni.2008.10.013 19084434

[92] Radic MZ Weigert M. Genetic and structural evidence for antigen selection of anti-DNA antibodies. Annu Rev Immunol. (1994) 12:487–520. 10.1146/annurev.iy.12.040194.002415 8011289

[93] Minegishi Y Conley ME. Negative selection at the pre-BCR checkpoint elicited by human mu heavy chains with unusual CDR3 regions. Immunity (2001) 14:631–41. 10.1016/S1074-7613(01)00131-5 11371364

[94] Keenan RA De Riva A Corleis B Hepburn L Licence S Winkler TH. Censoring of autoreactive B cell development by the pre-B cell receptor. Science (2008) 321:696–9. 10.1126/science.1157533 18566249

[95] Grimsholm O Ren W Bernardi AI Chen H Park G Camponeschi A Absence of surrogate light chain results in spontaneous autoreactive germinal centres expanding VH81X-expressing B cells. Nat Commun. (2015) 6:7077 10.1038/ncomms8077 25959489

[96] Ren W Grimsholm O Bernardi AI Hook N Stern A Cavallini N. Surrogate light chain is required for central and peripheral B-cell tolerance and inhibits anti-DNA antibody production by marginal zone B cells. Eur J Immunol. (2015) 45:1228–37. 10.1002/eji.201444917 25546233

[97] van Loo PF Dingjan GM Maas A Hendriks RW Surrogate-light-chain silencing is not critical for the limitation of pre-B cell expansion but is for the termination of constitutive signaling. Immunity (2007) 27:468–80. 10.1016/j.immuni.2007.07.018 17869135

[98] Sun L Kono N Shimizu T Toh H Xue H Numata O. Distorted antibody repertoire developed in the absence of pre-B cell receptor formation. Biochem Biophys Res Commun. (2018) 495:1411–7. 10.1016/j.bbrc.2017.11.171 29191653

[99] Aranburu A Hook N Gerasimcik N Corleis B Ren W Camponeschi A. Age-associated B cells expanded in autoimmune mice are memory cells sharing H-CDR3-selected repertoires. Eur J Immunol. (2017). 10.1002/eji.201747127. [Epub ahead of print]. 29266242

[100] Thorarinsdottir K Camponeschi A Gjertsson I Martensson IL CD21 -/low B cells: a snapshot of a unique B cell subset in health and disease. Scand J Immunol. (2015) 82:254–61. 10.1111/sji.12339 26119182

[101] Rubtsova K Rubtsov AV Cancro MP Marrack P. Age-associated B cells: a T-bet-dependent effector with roles in protective and pathogenic immunity. J Immunol. (2015) 195:1933–7. 10.4049/jimmunol.1501209 26297793 PMC4548292

[102] Faderl M Klein F Wirz OF Heiler S Alberti-Servera L Engdahl C. Two distinct pathways in mice generate antinuclear antigen-reactive B cell repertoires. Front Immunol. (2018) 9:16. 10.3389/fimmu.2018.00016 29403498 PMC5786517

[103] Reth M Nielsen P. Signaling circuits in early B-cell development. Adv Immunol. (2014) 122:129–75. 10.1016/B978-0-12-800267-4.00004-3 24507157

